# Plasma metabolomics and lipidomics signatures of motoric cognitive risk syndrome in community-dwelling older adults

**DOI:** 10.3389/fnagi.2022.977191

**Published:** 2022-09-07

**Authors:** Wanmeng Li, Xuelian Sun, Yu Liu, Meiling Ge, Ying Lu, Xiaolei Liu, Lixing Zhou, Xiaohui Liu, Biao Dong, Jirong Yue, Qianli Xue, Lunzhi Dai, Birong Dong

**Affiliations:** ^1^National Clinical Research Center for Geriatrics and Department of General Practice, State Key Laboratory of Biotherapy, West China Hospital, Sichuan University and Collaborative Innovation Center of Biotherapy, Chengdu, China; ^2^Department of Neurobiology, Care Sciences and Society, Division of Neurogeriatrics, Karolinska Institute, Solna, Sweden; ^3^School of Life Sciences, Tsinghua University, Beijing, China; ^4^Department of Medicine, Biostatistics, and Epidemiology, Division of Geriatric Medicine and Gerontology, School of Medicine, Johns Hopkins University, Baltimore, MD, United States

**Keywords:** motoric cognitive risk syndrome, pre-dementia, subjective cognitive complaint, slow gait speed, metabolomics and lipidomics, cross-sectional study

## Abstract

**Introduction:**

Motoric cognitive risk syndrome (MCR) is characterized by subjective cognitive complaints (SCCs) and slow gait (SG). Metabolomics and lipidomics may potentiate disclosure of the underlying mechanisms of MCR.

**Methods:**

This was a cross-sectional study from the West China Health and Aging Trend cohort study (WCHAT). The operational definition of MCR is the presence of SCCs and SG without dementia or mobility disability. The test and analysis were based on untargeted metabolomics and lipidomics, consensus clustering, lasso regression and 10-fold cross-validation.

**Results:**

This study enrolled 6,031 individuals for clinical analysis and 577 plasma samples for omics analysis. The overall prevalence of MCR was 9.7%, and the prevalence of MCR-only, assessed cognitive impairment-only (CI-only) and MCR-CI were 7.5, 13.3, and 2.1%, respectively. By consensus clustering analysis, MCR-only was clustered into three metabolic subtypes, MCR-I, MCR-II and MCR-III. Clinically, body fat mass (OR = 0.89, CI = 0.82–0.96) was negatively correlated with MCR-I, and comorbidity (OR = 2.19, CI = 1.10–4.38) was positively correlated with MCR-III. Diabetes mellitus had the highest ORs above 1 in MCR-II and MCR-III (OR = 3.18, CI = 1.02–9.91; OR = 2.83, CI = 1.33–6.04, respectively). The risk metabolites of MCR-III showed relatively high similarity with those of cognitive impairment. Notably, L-proline, L-cystine, ADMA, and N1-acetylspermidine were significantly changed in MCR-only, and PC(40:3), SM(32:1), TG(51:3), eicosanoic acid(20:1), methyl-D-galactoside and TG(50:3) contributed most to the prediction model for MCR-III.

**Interpretation:**

Pre-dementia syndrome of MCR has distinct metabolic subtypes, and SCCs and SG may cause different metabolic changes to develop MCR.

## Introduction

Dementia is a chronic disease with high medical cost-burdens to society and now ranks as the 7th leading cause of mortality worldwide (Gauthier et al., 2021). Approximately 55 million people are suffering from dementia, which is estimated to increase to 152 million in 2050 (Gauthier et al., 2021), although less than 25% globally are diagnosed, especially as low as approximately 10% diagnosis rates in the lower-income countries (Semba et al., 2020; Gauthier et al., 2021). Dementia has a long preclinical stage of several years to decades before subtle cognitive alterations are detectable (Semba et al., 2020). Early identification of the increased risk individuals for future dementia, may offer timely treatment, since disease-modifying strategies for dementia like Alzheimer’s disease are almost unavailable worldwide. Motoric cognitive risk syndrome (MCR), characterized by subjective cognitive complaints (SCCs) and slow gait speed (SG) in the absence of mobility assistance and dementia, was first proposed and validated as a pre-dementia syndrome in 2013 by Verghese et al. (2012, 2014). The prevalence of MCR was 10% in older adults worldwide (Maggio and Lauretani, 2019; Meiner et al., 2020).

Motoric cognitive risk syndrome has received increasing attention (Verghese et al., 2012, 2013; Ayers and Verghese, 2016; Wang et al., 2016; Maguire et al., 2018), and previous studies provide figures and data further justifying this pre-dementia syndrome. In a study consisting of 26,802 samples has showed that MCR increases two-fold incidence of cognitive impairment (aHR 2.0) (Verghese et al., 2014). MCR increases the incidence of falls and post-fall fractures (Beauchet et al., 2019), hospitalization, disability (Doi et al., 2017), and mortality in older adults (Yuan et al., 2021). MCR patients have poorer performance in global cognitive performance by the Mini-Mental Status Examination, Free and Cued Selective Reminding Test, Frontal Assessment Battery and Trail Making Test parts A and B (Sekhon et al., 2017; Maguire et al., 2018). The risk factors of MCR include aging, low education, cardiovascular diseases, obesity, physical inactivity, anxiety-depressive disorders (Meiner et al., 2020), small cerebral vessel diseases (Wang et al., 2016), and frailty (Sathyan et al., 2019). Increased levels of interleukin-6 and C-reactive protein have also been observed in MCR patients (Bortone et al., 2021). Compared with healthy controls, MCR patients have lower gray matter volume in the prefrontal and premotor cortexes, and higher levels of lacunar lesions in the frontal lobe (Sekhon et al., 2019), which are related to motor planning and modulation (Blumen et al., 2019).

Previous studies indicated that the dysregulation of specific lipids and amino acids is associated with cognitive declines (Semba et al., 2020). However, the metabolomics and lipidomics disorders caused by MCR is still less studied. Here, we performed plasma metabolomics and lipidomics investigations on MCR, preliminarily providing insights into MCR metabolic mechanisms and signatures.

## Materials and methods

### Study design of the West China Health and Aging Trend cohort study and the sample selection

This was a cross-sectional study, and the data came from the ongoing prospective West China Health and Aging Trend cohort study (WCHAT), which was initiated in 2018. The Ethical Review Committee of West China Hospital approved WCHAT [Permission number: 2017(445)], and it was registered on the Chinese Clinical Trial Registry [ChiCTR1800018895] (Hou et al., 2021). All procedures were conducted along the principles of the 1964 Declaration of Helsinki guidelines and its amendments. In brief, WCHAT originally recruited 7,536 community-dwelling residents. Most of the participants were over 50 years old, 37.47% were males, and 62.53% were females.

Specifically, the WCHAT study recruited 7,536 community residents. After excluding 60 persons under 50 years old, 22 people with dementia or severe cognitive impairment, 617 individuals with ADL assistance and 806 persons with missing MCR diagnostic information, 6,031 participants were finally retained for subsequent epidemiological analysis. This study included 577 plasma samples for metabolomics analysis from participants who were willing to attend the follow-up visit, and the subjects with severe diseases were excluded. Moreover, to ensure the reliability of the metabolomics and lipidomics analysis, we recruited as many subjects with MCR as possible.

### Evaluation of motoric cognitive risk syndrome

The operational definition of MCR was the presence of SCCs and SG but without dementia or mobility disability, as proposed and validated by Verghese et al.’s (2012, 2014), Maggio and Lauretani (2019), and Stephan et al. (2020). The diagnosis of present MCR was described elsewhere in detail (Sun et al., 2022). Dementia was identified by self-and/or proxy-reported previous diagnoses by physicians. Additionally, we excluded probable dementia which was classified as severe cognitive impairment using the 10-item Short Portable Mental Status Questionnaire (SPMSQ) (Pfeiffer, 1975). Mobility disability was defined by Activities of Daily Living (ADLs) assistance. The SCCs were based on standardized questionnaire items: (1) the Geriatric Depression Scale (GDS): “Do you feel that you have more problems with memory than most?” (endorsed response: yes) (Stephan et al., 2020); (2) In the past month, have memory problems affected your daily activities? (endorsed response: yes). Gait speed was obtained by measuring the normal pace of walking speed over 4 m. SG was determined by having a walking speed greater than or equal to 1 standard deviation (SD) below the average of age- and sex-specific values, to overcome population and program variability (Capistrant et al., 2014). The present cognitive impairment group (CI) was defined as having mild or moderate cognitive impairment by the SPMSQ (Pfeiffer, 1975).

### Metabolomics and lipidomics

Trained professionals collected three tubes of fasting peripheral blood from participants in the morning. Routine blood tests were performed on the same day. The plasma samples were centrifuged at 14,000 g at 4°C for 20 min, and the supernatants were used to extract hydrophilic metabolites and lipids. ^13^C_6_ L-Lysine hydrochloride powder (Silantes) and ^13^C_6_^15^N_4_ L-Arginine hydrochloride powder (Silantes) were used to monitor the extraction efficiency of hydrophilic metabolites. PE (16:0-D31-18:1) was used to monitor the extraction efficiency of lipids. The hydrophilic metabolites were extracted with pre-cooled methanol (Yuan et al., 2012), while the lipids were extracted according to the method of Bligh and Dyer (1959).

Untargeted metabolomics and lipidomics were carried out at the Facility Center of Metabolomics and Lipidomics of Tsinghua University (Patti et al., 2012; Hakimi et al., 2016; Tang et al., 2016). A BEH amide column (Waters, United States) and a BEH C18 column (Waters, United States) were used for metabolomics analysis under positive and negative ion modes, respectively. A CORTECS C18 column (Waters, United States) was used for lipidomics under positive mode. Pooled quality controls (QCs) were inserted for every 15–20 injections of plasma samples. Polar metabolites were assigned using Tracefinder (Thermo, CA, United States) based on an in-house database. Standard MS/MS spectra of over 1,500 metabolites were included in the database. Lipids were identified using Lipidsearch (Thermo, CA, United States) software. Only lipids with reliable MS/MS were used for the following statistical analyses.

### Bioinformatics analysis

The metabolites and lipids were extracted in the same batch. Moreover, the samples were continuously analyzed by mass spectrometry. Some metabolites were deleted, including those with missing values NA >20% and CV (Coefficient of Variation of QC samples) >30%. To normalize the metabolite intensity, we first calculated the average total intensity of all samples, and then divided the total intensity of each sample by the average total intensity of all samples to get a coefficient. Finally, the measured intensity of each metabolite was divided by the coefficient for the corresponding sample to obtain the normalized intensity of each metabolite in that sample. The lipidome and the metabolome data obtained in positive acquisition mode, and the metabolome data obtained in negative acquisition mode, were filled the blank with half of the minimum intensity of metabolites in all samples. The intensity values were log2-transformed to reduce skewness and stabilize the variance. Statistical analyses were conducted using R version 4.1.0 or SPSS software version 26 (IBM Corporation, Chicago, IL, United States).

The Kolmogorov-Smirnov test was used to test the normal distribution of the continuous variables, while the Mann–Whitney *U* test was utilized for difference analyses and the median ratio value was calculated, when the metabolomic and lipid data were abnormally distributed (*p* < 0.05). Logistic regression was used for risk analysis adjusted for sex and age. Linear regression was used for the correlation of continuous variables. The Kruskal-Wallis test was used for the comparison of multiple groups. Consensus clustering of metabolomics and lipidomics data was carried out to determine the metabolic subtypes of participants with MCR (R package: ConsensusCluster Plus). Lasso was used to select metabolic characteristics and establish a regression model to predict MCR-III. A 10-fold cross-validation was performed, and the minimum lambda value plus standard error was selected as the best lambda to solve the model overfitting problem (R package: glmnet, *s* = lambda.1 s). The contribution of each metabolite in each prediction model was defined by the coefficient: Contribution = abs(coefficient)/sum[abs(coefficient)] (Liang et al., 2020).

## Results

### Epidemiological and clinical features of motoric cognitive risk syndrome

**FIGURE 1:**
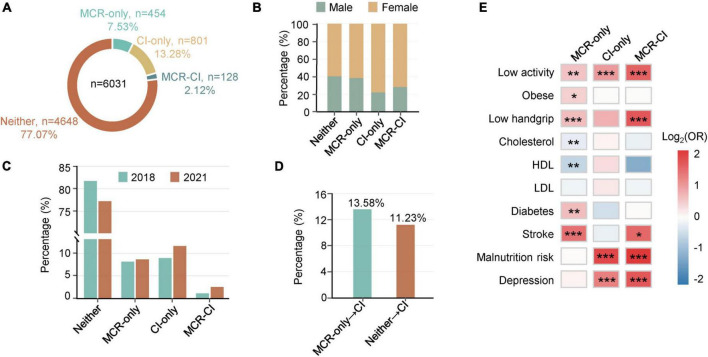
Epidemiological statistics of motoric cognitive risk, cognitive functional impairment and comorbid motoric cognitive risk-cognitive functional impairment. **(A)** A statistical analysis of the West China Health and Aging Trend study (WCHAT). 7.53% MCR-only, 13.28% CI-only, 2.12% MCR-CI participants in WCHAT (*n* = 6,031). **(B)** Percentage of men and women in Neither, MCR-only, CI-only, MCR-CI. **(C)** Heatmaps showing associated factors for MCR, CI, and MCR-CI. Red represents positive risk and blue represents negative risk. Significance is indicated by “*”(*0.01 < *p* < 0.05, **0.001 < *p* ≤ 0.01, ***p ≤ 0.001). **(D)** A statistical analysis of 1983 participants with MCR-only, CI-only, and MCR-CI in 2018 and 2021. **(E)** Incidence comparison between MCR-only and Neither for CI in the fourth year of follow-up.

The overall prevalence of MCR was 9.7% (*n* = 582), while 15.4% (*n* = 929) of participants suffered from CI. In the clinical subgroups, 454 participants (7.5%) were diagnosed with MCR-only, 801 (13.3%) with CI-only, 128 (2.1%) with concurrent MCR and CI (MCR-CI), and 4,648 participants (77.1%) without MCR or CI (Neither) (Figure 1A). Overall, CI patients constituted 22.0% of MCR, while MCR patients accounted for 13.8% of CI, which were in line with other studies (Sekhon et al., 2017). In demography, the mean ages sequentially increased among Neither (61.7 ± 8.1), MCR-only (64.1 ± 7.7), CI-only (64.4 ± 8.9) and MCR-CI (66.9 ± 9.1) groups, while the marriage rates successively decreased. Female numbers prevailed in the four groups (Figure 1B and Supplementary Table 1). The prevalence of MCR and CI significantly differed by ethnicity (Supplementary Table 1) (Liu et al., 2020). Additionally, the distribution of a group of biological and clinical factors was significantly divergent among the Neither, MCR-only, CI-only and MCR-CI groups (Supplementary Table 1). Multiple logistic regressions adjusted for age and sex were further analyzed. The significantly correlated factors with specific subgroups differed among the MCR-only, CI-only and MCR-CI groups. Low activity, obesity, low handgrip strength, diabetes mellitus and stroke were positively associated with MCR-only, while cholesterol and high-density lipoprotein (HDL) were negatively associated with it. For CI-only, it had positive associations with low activity, malnutrition risk, and depression. Similar to CI-only, we found that MCR-CI was positively correlated with low activity, low handgrip strength, malnutrition risk and depression (Figure 1C), in agreement with the previous reports (Beauchet et al., 2020).

To verify whether MCR had a higher incidence of CI than the healthy Neither group, we partially completed the 4-year follow-up of WCHAT in 2021, and 1983 participants were available for epidemiological analysis. We found that the prevalence of MCR-only, CI-only, and MCR-CI was higher in 2021 than in 2018 (Figure 1D). Moreover, the incidence of CI (13.58%) in MCR-only people was higher than that (11.23%) in Neither group, which was initially healthy people in 2018 (Figure 1E).

### Metabolic characterization of motoric cognitive risk syndrome

**FIGURE 2:**
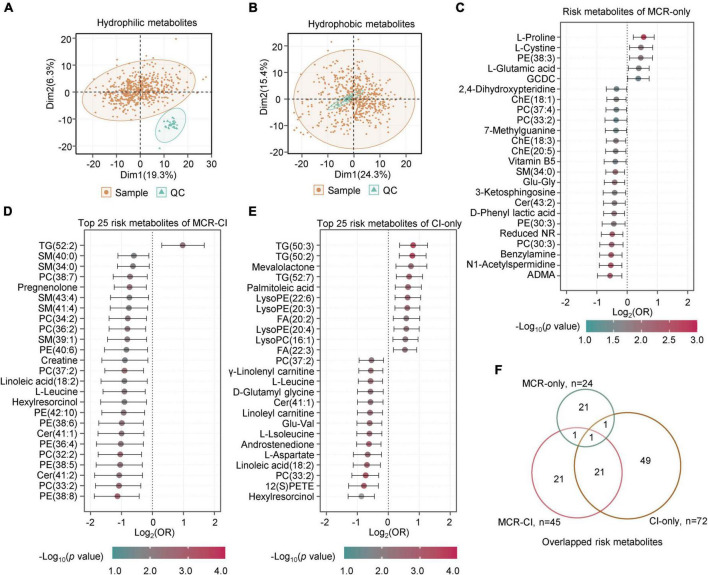
The metabolites with significant differences in motoric cognitive risk, cognitive functional impairment and comorbid motoric cognitive risk-cognitive impairment. **(A)** PCA of the metabolome of samples and quality controls (QCs). **(B)** PCA of the lipidome of samples and QCs. **(C)** The forest map showing the risk metabolites of MCR-only by logistic regression analysis (*p* < 0.05). There were 5 positive risk metabolites and 19 negative risk metabolites. **(D)** The forest map showing the top 25 risk metabolites of MCR-CI by logistic regression analysis (*p* < 0.05). There was 1 positive risk metabolite and 24 negative risk metabolites. **(E)** The forest map showing the top 25 risk metabolites of CI-only by logistic regression analysis (*p* < 0.05). There were 11 positive risk metabolites and 14 negative risk metabolites. **(F)** Overlapping analysis of risk metabolites of MCR-only, CI-only and MCR-CI. The metabolites in panels **(C–E)** are ranked by the crude odds ratios (OR). The segment of each metabolite means 95% CI. Cer, ceramide; PC, phosphatidylcholine; LysoPC, lysophosphatidylcholine; PE, phosphatidylethanolamine; LysoPE, lysophosphatidylethanolamine; TG, triacylglycerol; SM, sphingomyelins; FA, fatty acid; ChE, cholesterol ester; GCDC, glycochenodeoxycholic acid; ADMA, asymmetric dimethylarginine; Reduced NR, 1-(beta-D-ribofuranosyl)-1,4-dihydronicotinamide.

To investigate the metabolic characteristics of MCR, metabolomics and lipidomics profiling were carried out in 577 plasma samples, with 82 in MCR-only, 66 in CI-only, and 19 in MCR-CI. In total, we identified 345 hydrophilic metabolites and 231 lipids. PCA of the metabolome and lipidome data of the samples and the QCs showed high data quality (Figures 2A,B). Logistic regression analysis of the metabolites after adjusting for sex and age identified 24, 72, and 45 differential metabolites in the MCR-only, CI-only, and MCR-CI groups, respectively (*p* < 0.05). All risk metabolites of MCR-only and the top 25 risk metabolites of CI-only and MCR-CI were shown in Figures 2C–E and Supplementary Tables 4–6. L-Proline (OR = 1.46, CI = 1.15–1.85), and L-cystine (OR = 1.37, CI = 1.06–1.79) were positively associated with MCR-only, while asymmetric dimethylarginine (ADMA) (OR = 0.67, CI = 0.51–0.88), and N1-acetylspermidine (OR = 0.69, CI = 0.53–0.88) were negatively associated with it. Triglycerides (TG, 50:2) (OR = 1.71, CI = 1.27–2.32) and hexylresorcinol (OR = 0.55, CI = 0.40–0.73) had the highest and the lowest ORs for CI-only, respectively. For MCR-CI, TG (52:2) was the only positively correlated, and PE(38:3) had the lowest OR. Further overlapping analysis showed that the risk metabolites of MCR-only were different from those of CI-only and MCR-CI, but the metabolic changes induced by MCR-CI and CI-only were relatively closer (Figure 2F). This indicated that MCR-only had a distinct metabolic profile.

### Metabolic stratification of motoric cognitive risk syndrome

Omics studies show that the same diagnosed diseases may have distinct molecular profiles (Mapstone et al., 2014). To identify whether MCR-only has distinguishing metabolic subtypes, we performed unsupervised clustering analysis using the top 25% most variable metabolites (144 metabolites) from 576 metabolites of 82 MCR-only participants. Consequently, three subtypes, MCR-I, MCR-II, and MCR-III were identified. Multiple logistic regression further verified the clinical discrepancy of these metabolic MCR subtypes compared with healthy participants without MCR and CI (Supplementary Table 3). The three subtypes shared five factors with the ORs all below 1, including body mass index (BMI), short physical performance battery (SPPB) score, skeletal muscle mass, total body water and body minerals. Among them, the factor of body minerals had the highest protective association with these three subtypes, with ORs all below 0.3. Body fat mass (OR = 0.89, CI = 0.82–0.96) was negatively correlated with MCR-I, and comorbidity (OR = 2.19, CI = 1.10–4.38) was positively correlated with MCR-III. Diabetes mellitus had the highest ORs for MCR-II (OR = 3.18 CI = 1.02–9.91) and MCR-III (OR = 2.83 CI = 1.33–6.04) but it was not significant for MCR-I.

**FIGURE 3:**
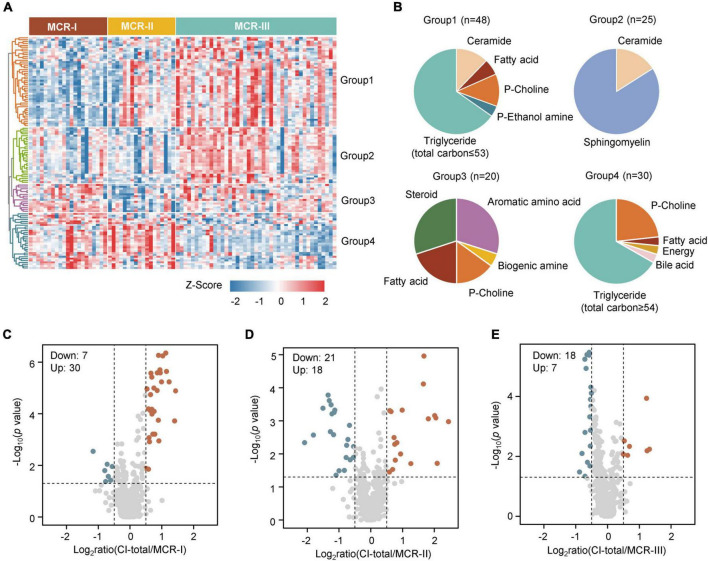
Three metabolic subtypes of MCR. **(A)** The heatmap shows the relative abundance (z score transformed) of the significantly changed metabolites in three clusters (Kruskal-Wallis test, FDR <0.05). **(B)** Classification of metabolites into four groups. **(C)** The significantly changed metabolites in both CI-total and MCR-I (Mann–Whitney *U* test, *p* < 0.05, Log_2_ratio >0.5 or Log_2_ratio <–0.5). **(D)** The significantly changed metabolites in both CI-total and MCR-II (Mann–Whitney *U* test, *p* < 0.05, Log_2_ratio >0.5 or Log_2_ratio < –0.5). **(E)** The significantly changed metabolites in both CI-total and MCR-III (Mann–Whitney *U* test, *p* < 0.05, Log_2_ratio >0.5 or Log_2_ratio <–0.5).

To identify metabolic disparities among the three subtypes, we screened out the differentially changed metabolites using the Kruskal-Wallis test. A total of 124 metabolites were significantly different among the three subtypes (False Discovery Rate (FDR) <0.05) (Figure 3A), and consensus clustering was used to divide them into four groups according to the patterns of changes. The levels of triglycerides, which occurred mainly in group 1 and group 4, were reported to be closely associated with cognitive regulation (Figure 3B) (van der Lee et al., 2018). In particular, triglycerides in group 1 were highly expressed in MCR-III (Figure 3A), and the total carbon number of most of these triglycerides was less than 53 (Figure 3B). In contrast, triglycerides in group 4 were highly expressed in MCR-I and MCR-II (Figure 3A), and the total carbon numbers of most of them were above 54 (Figure 3B). Besides, more triglycerides, such as TG(56:9), TG(58:5), and TG(58:6), contained unsaturated side chains in group 4, which is consistent with previous findings that long-chain polyunsaturated triglycerides (PUTGs) were significantly reduced in the precursor stage of mild cognitive impairment (MCI) and the reduction in PUTGs may be related to the early changes in AD (Bernath et al., 2020). Group 2 was higher in MCR-III, with metabolites including sphingomyelin and ceramides. Elevated serum sphingomyelin and ceramide levels have been reported to be associated with an increased risk of AD (Wong et al., 2017). Aromatic amino acids and steroids accounted for a large proportion in group 3 (Figure 3B).

### Identification of the most harmful motoric cognitive risk syndrome subtype and metabolites

To identify the metabolic characteristics unique to the three subtypes, especially MCR-III, we performed logistic regression analysis within the MCR subtypes. Taking the healthy Neither group as a reference, we identified 65 risk metabolites for MCR-I, 75 for MCR-II, and 74 for MCR-III (Supplementary Tables 7–9).

To determine the relationship between MCR subtypes and cognitive impairment, we, respectively screened the metabolites showing differences between MCR subtypes and CI-total (which contains MCR-CI and CI-only) using the Mann–Whitney *U* test. The metabolic and lipidomic features of MCR-III were relatively more correlated with CI-total than MCR-I and MCR-II (Figures 3C–E), suggesting that metabolic MCR-III may exacerbate cognitive decline and dementia more than other subtypes.

**FIGURE 4:**
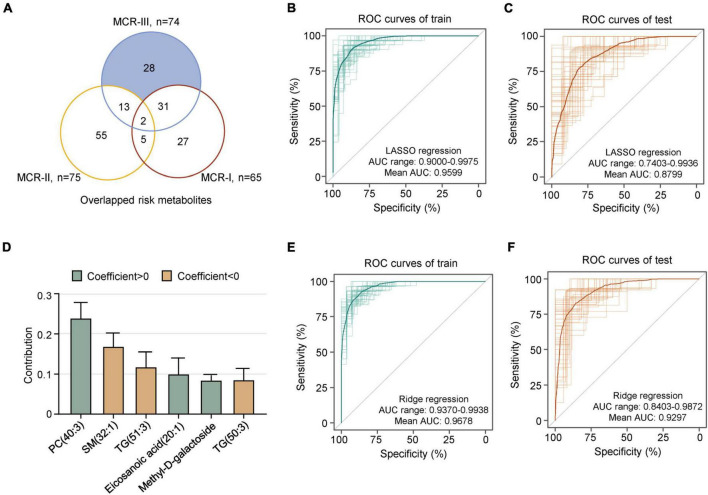
A metabolic model predicts MCR-III. **(A)** Overlapping risk metabolites of MCR-I, MCR-II and MCR-III. Shaded in blue are the risk metabolites specific to MCR-III, which were used in subsequent modeling. **(B)** The 50 ROC curves of the training sets in lasso regression; the bold one is the mean AUC of 50 times. **(C)** The 50 ROC curves of the test sets in lasso regression; the bold one is the mean AUC of 50 times. **(D)** Contribution of the six metabolites to the prediction model of MCR-III or no. The error bar represents a 95% CI. **(E)** The 50 ROC curves of the training sets in ridge regression; the bold one is the mean AUC of 50 times. **(F)** The 50 ROC curves of the test sets in ridge regression; the bold one is the mean AUC of 50 times.

Overlapping analysis showed that 28 risk metabolites were unique to the MCR-III subtype (Figure 4A). Next, we developed a specific metabolic model to identify MCR-III participants out of all MCR individuals. A random sampling of 50 discovery sets (70% of samples) with replacement, and feature selection from 28 metabolic features unique to MCR-III, were used to build LASSO regression models, which showed the best 10-fold cross-validation performance for a given phenotype in the cohort. We ran the model built in the discovery sets with the remaining MCR participants as verification sets (*n* = 25), to measure the independent performance of the metabolic model. Among the 50 random metabolic models, the mean receiver operating characteristic (AUROC) of the discovery sets was 0.9599 (AUC range: 0.9000–0.9975), and the mean AUROC of the verification sets was 0.8799 (AUC range: 0.7403–0.9936) (Figures 4B,C).

When the value of λ was one standard error plus the minimum value, we analyzed the contribution of these metabolites in the model. The metabolites of PC(40:3), SM(32:1), TG(51:3), eicosanoic acid(20:1), methyl-D-galactoside and TG(50:3) performed well and contributed robustly in most of the 50 models (Figure 4D). Thereby, these six metabolites can be used as key metabolites to distinguish MCR-III from other MCRs. Then, we used them as eigenvalues to establish a ridge regression model to predict MCR-III and not MCR-III. As above, 50 different discovery sets were randomly selected to build 50 models. We found that the accuracy of the model’s predictions was greatly improved. The mean AUROCs value of both discovery sets and verification sets was above 0.9, and the best AUROC value was above 0.98 (Figures 4E,F).

### Metabolic features associated with subjective cognitive complaint and slow gait

**FIGURE 5:**
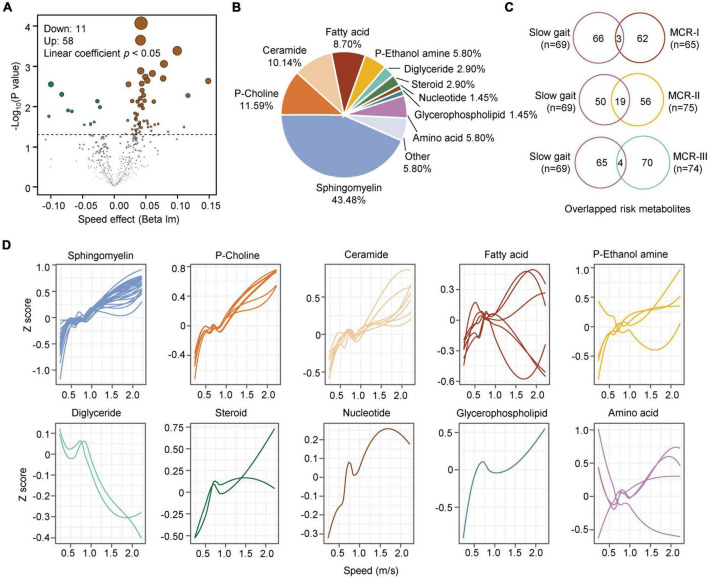
Metabolic characteristics associated with slow gait. **(A)** The volcano map shows the metabolites associated with slow gait (linear coefficient, *p* < 0.05). **(B)** Classification of the significantly changed metabolites with step speed. **(C)** Overlap analysis of metabolites associated with slow gait and those associated with MCR-I, MCR-II, and MCR-III (logistic regression, *p* < 0.05). **(D)** The correlations between slow gait and 10 classes of gait speed-associated metabolites are plotted with a Loess curve.

Subjective cognitive complaints and SG are two key components used to evaluate MCR. We assumed that the MCR subtypes might have metabolic features similar to those of SCCs and/or SG. To determine the association between MCR subtypes and SG, we screened metabolites closely related to walking speed. Linear regression analysis was adjusted for sex and age. A total of 69 metabolites changed significantly with the alteration of gait speed, of which 58 were positively correlated and 11 were negatively correlated with gait speed (Figure 5A). The 69 risk metabolites of SG could be classified into ten categories (Figure 5B). Among them, sphingomyelins, p-choline, ceramides, steroids, glycerophospholipids and nucleotides increased with speed, while diglycerides decreased with speed (Figure 5D). Overlapping analysis revealed the largest number of common risk metabolites between SG and MCR-II (Figure 5C). Notably, four risk metabolites of SG, Hex1Cer(41:1), SM(38:3), SM(36:0), and SM(32:1), were also risk metabolites of MCR-III.

**FIGURE 6:**
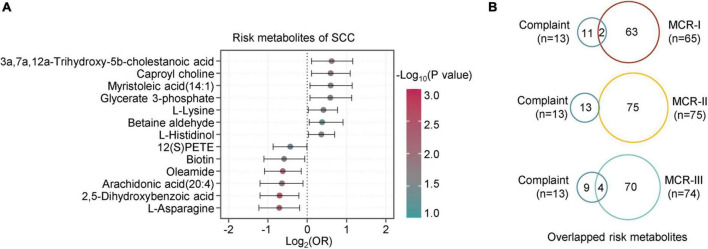
Metabolic characteristics associated with subjective cognition complaints. **(A)** The forest figure shows the metabolites associated with cognitive complaints (logistic regression, *p* < 0.05). The metabolites are ranked by the crude odds ratios (OR). The segment of each metabolite means 95% CI. **(B)** Overlap analysis of metabolites associated with complaints and those associated with MCR-I, MCR-II, and MCR-III (logistic regression, *p* < 0.05).

Similarly, the logistic regression analysis revealed 13 metabolites associated with SCCs, of which seven were positive and six were negative correlations (Figure 6A). When comparing them with the metabolites of MCR subtypes, we found two overlapping metabolites between SCC and MCR-I, none between SCCs and MCR-II, and four between SCCs and MCR-III (Figure 6B). In detail, these four compounds were 2,5-dihydroxybenzoic acid, oleamide, arachidonic acid(20:4) and myristoleic acid (14:1). It is well known that subjective cognitive complaints (SCCs) are currently considered a major feature of mild cognitive impairment (MCI) (Mitchell, 2008). However, only three common risk metabolites of CI and SCCs have been found. We assume that SCC may not be severe enough to cause significant metabolic alterations. As a result, we only obtained 13 risk metabolites for SCC. In contrast, 75 risk metabolites were obtained for CI.

## Discussion

Motoric cognitive risk syndrome, with the two components SCCs and SG, is a stronger predictor of the cognitive decline and dementia than either measure alone (Verghese et al., 2014). Subjective cognitive complaints (SCCs) and slow gait speed (SG) are two early indications of cognitive decline and dementia (Semba et al., 2020). SCCs probably precedes MCI by up to 15 years (Reisberg et al., 2008), while the occurrence of the decline in gait speed is 12 years ahead of MCI (Buracchio et al., 2010). The pooled hazard ratios (HR) of MCR were 1.5 to 2.7 for cognitive impairment and 1.9 to 3.27 for dementia (95% CI, 1.75–2.39) (Verghese et al., 2012, 2014), but not all MCR will develop into MCI, dementia, or even AD (Semba et al., 2020). It has been reported that the prevalence of MCR varied in different countries and/or regions, with 8.0% in Europe, 6.3% in Japan and 7.0% in United States (Maggio and Lauretani, 2019). The overall MCR prevalence of 9.7% in the present study was in line with the pooled global prevalence of 9.7% estimated from 26,802 participants across 17 countries (Verghese et al., 2014).

Metabolomic platforms potentiate the detection of hundreds of metabolites for the discovery of disease phenotypes. However, multi-omics platforms have been barely used for MCR investigation, which suggests the application of these approaches to reveal the pathobiological mechanism of MCR. Metabolomic investigations provided a number of clues in identifying specific amino acids and lipids for the prediction of cognitive decline (Li et al., 2019; Semba et al., 2020). Additionally, early identification of the subpopulation of MCR with the tendency of developing cognitive impairment, dementia, or AD provides opportunities to give timely preventive strategies (Verghese, 2021). Notably, MCR shares connections but also has synergistic discrepancies with other cognitive impairment syndromes, such as MCI, and the causal-effective association between them remains to be elucidated (Semba et al., 2020; Cheng et al., 2021). To exclusively focus on MCR-only individuals, we rationally divided the population into four groups, Neither, MCR-only, CI-only, and MCR-CI, and this grouping method was verified by disparities in both clinical and metabolic characteristics (Figures 1, 2).

Targeting MCR-only participants, we verified the plasma metabolome and lipidome of MCR based on a large multi-center cohort study in China. First, MCR was classified into three distinct metabolic subtypes: MCR-I, MCR-II and MCR-III. Those individuals with the MCR-III subtype were more likely to develop CI than the others, followed by MCR-II and MCR-I. As the present results indicated that MCR-III was the most striking metabolic subtype among the three, we further explored the predictive models and determined the best-performing one with a model AUROC above 0.9, showing a good predictive performance (Figures 4B,C). More precisely, the model was composed of the six metabolites which were used as key markers to distinguish MCR-III from other MCRs. Thus, we assumed that the present findings supported the reasonable stratification of MCR.

Regarding the metabolic and lipidomic characteristics of the two components of MCR, overlapping result discovered that a larger number of common risk metabolites between SCCs and MCR-III, while the SCCs people patients tend to develop cognitive impairment (Semba et al., 2020). A previous multi-center study showed that SCCs rather than SG contributes more to the progression of dementia after the diagnosis of MCR (Verghese et al., 2019). The metabolic alterations of SCCs and SG justified our stratification of MCR and strengthened the previous assumption that MCR-III was mostly related to cognitive deterioration.

To our knowledge, our findings are the first to provide an overview of the metabolic and lipidomic profile of the pure MCR population and favor previous verification that plasma metabolites are associated with cognitive aging and cognitive decline (Ackerman et al., 2018; Bernath et al., 2020; Lefèvre-Arbogast et al., 2021). Triglycerides are significant in maintaining the homeostasis of specific fatty acids. When triglycerides are disrupted by inner and outer damaging stimulators, toxic saturated lipids accumulate, causing overproduction of toxic acyl-carnitines, and saturated ceramides, and activation of the NF-κB pathway (Ackerman et al., 2018). Specifically, the level of long-chain polyunsaturated triglycerides significantly reduced in the precursor stage of MCI and dementia (Bernath et al., 2020). In this study, triglycerides with saturated side chains increased in the MCR-III subtype, while the triglycerides with unsaturated side chains manifested the opposite changes.

Disorders of plasma phospholipids were reported in predicting antecedent cognitive impairment in older adults (Mapstone et al., 2014; Toledo et al., 2017). Phosphatidylcholine (PC) is an important class of lipids for cognitive health. Reduced PC species, such as PC(33:2), PC(34:2), PC(35:2), PC(36:2), PC(37:2), and PC(34:3), showed the association with the loss of cognitive function (Shea, 2019). A group of 10 plasma lipids were identified, and the level of PCs and acylcarnitine significantly reduced in participants who developed amnesic MCI or AD within a 2 to 3-year time frame (Mapstone et al., 2014). A longitudinal study found that PC(16:0_18:2), PC(18:0_18:1), and PC(18:1_18:1) were positively correlated with the performance of global and specific cognitive domains. Among cognitively unimpaired older individuals, PC (14:0_14:0) was independently associated with slower cortical thinning and amyloid deposition (Li et al., 2019). MCR is a pre-dementia syndrome, pathologically with lower overall cortical thickness and regional gray matter volume (Beauchet et al., 2016; Blumen et al., 2017). In our study, a low level of PC(40:3) is a striking feature to identify the MCR-III subtype. Additionally, PC(40:3) was one of the six key metabolites in the prediction model to distinguish MCR-III from other MCRs.

In addition, our results shared concordance with previous findings. For example, B vitamins slow the course of cognitive decline (Smith et al., 2018). Palmitoleic acid, myristoleic acid, and alpha-linolenic acid were all reported to be closely correlated with cognition (Varma et al., 2018; Wang et al., 2020). Compared with the control group, the serum level of linoleic acid, myristic acid, and palmitic acid decreased in MCI and AD patients.

However, there are still some limitations to this study. The main body of our metabolic and lipidomic findings was from one-time collected biological samples based on an ongoing longitudinal multi-center cohort; therefore, we accessed limited causal-effective evidence. In addition, because the number of MCRs was not large enough, most of the MCR-related metabolites were not significant when multiple testing correction using False Discovery Rate (FDR) was carried out. Therefore, most of our tests used raw *p*-values. Although the analysis of metabolic and lipidomic data adjusted some covariables, the comorbidities in the data analysis such as sleep disorders and depressive and diabetes mellitus, would benefit future investigations.

Motoric cognitive risk syndrome in the pre-dementia phase has distinct metabolic subtypes, and SCC and SG display discordant metabolic features in developing MCR. The pathogenesis and mechanism of MCR need further investigations.

## Data availability statement

The original contributions presented in this study are included in the article/Supplementary material, further inquiries can be directed to the corresponding authors.

## Ethics statement

The studies involving human participants were reviewed and approved by the Ethical Review Committee of West China Hospital. The patients/participants provided their written informed consent to participate in this study.

## Author contributions

XS, WL, QX, LD, and BrD: conception and design of the study. XS, YLi, MG, YLu, LZ, XlL, XhL, BD, JY, and QX: acquisition and analysis of data. WL, XS, LD, and BrD: drafting a significant portion of the manuscript and figures. All authors contributed to the article and approved the submitted version.
