# Supplementary material for: Plasma metabolomics and lipidomics signatures of motoric cognitive risk syndrome in community-dwelling older adults
Source: Front Aging Neurosci. 2022 Sep 7;14:977191. doi: 10.3389/fnagi.2022.977191 (PMC9490321; doi:10.3389/fnagi.2022.977191)
Supplement: Supplementary file 1 [file Data_Sheet_1.docx]

# Table S1. Distributions of demographic and clinical characteristics of motoric cognitive risk syndrome and cognitive impairment status.(n=6031)

| Characteristics | Neither  (N=4648) | MCR-only  (N=454) | CI-only  (N=801) | MCR-CI  (N=128) | *p* value |
| --- | --- | --- | --- | --- | --- |
| Age ^a^ | 61.7±8.1 | 64.1±7.7 | 64.4±8.9 | 66.9 ±9.1 | <0.001 |
| Female ^b^ | 2774 (59.7%) | 280 (61.7%) | 625 (78.0%) | 92 (71.9%) | <0.001 |
| Marital status ^b^ |  |  |  |  | <0.001 |
| Married | 3945 (84.9%) | 378 (83.3%) | 610 (76.2%) | 87 (68.0%) |  |
| Unmarried/Widowed/Divorced | 703 (15.1%) | 76 (16.7%) | 191 (23.8%) | 41 (32.0%) |  |
| Ethnicity ^b^ |  |  |  |  | <0.001 |
| Han | 1928 (41.5%) | 210 (46.3%) | 180 (22.5%) | 43 (33.6%) |  |
| Qiang | 856 (18.4%) | 37 (8.1%) | 146 (18.2%) | 11 (8.6%) |  |
| Tibetan | 874 (18.8%) | 129 (28.4%) | 181 (22.6%) | 46 (35.9%) |  |
| Yi | 338 (7.3%) | 41 (9.0%) | 134 (16.7%) | 18 (14.1%) |  |
| Uighur | 439 (9.4%) | 25 (5.5%) | 74 (9.2%) | 3 (2.3%) |  |
| Others | 213 (4.6%) | 12 (2.6%) | 86 (10.7%) | 7 (5.5%) |  |
| Education ^b^ |  |  |  |  | <0.001 |
| Illiterate | 975 (21.0%) | 146 (32.2%) | 448 (55.9%) | 74 (57.8%) |  |
| Primary school | 1648 (35.5%) | 182 (40.1%) | 191 (23.8%) | 35 (27.3%) |  |
| Middle school | 1139 (24.5%) | 72 (15.9%) | 68 (8.5%) | 10 (7.8%) |  |
| Secondary school and above  above | 886 (19.1%) | 54 (11.9%) | 94 (11.7%) | 9 (7.0%) |  |
| BMI ^a^ | 25.4±4.1 | 25.9±4.4 | 25.0±4.9 | 24.9±4.3 | 0.002 |
| Obese (yes)^b^ | 1107 (23.8%) | 128 (28.2%) | 186 (23.2%) | 28 (21.9%) | 0.170 |
| Low handgrip (yes) ^b^ | 1903 (40.9%) | 254 (55.9%) | 468 (58.4%) | 93 (72.7%) | <0.001 |
| 4-Meter gait speed (m/s) ^a^ | 0.9±0.2 | 0.5±0.1 | 0.8±0.2 | 0.4±0.1 | <0.001 |
| Skeletal Muscle Mass (kg) ^a^ | 22.3±4.4 | 21.5±4.5 | 20.4±4.0 | 20.1±4.1 | <0.001 |
| Body Fat Mass (kg) ^a^ | 21.2±6.9 | 22.0±7.5 | 21.0±7.8 | 20.3±8.0 | 0.094 |
| Total Body Water (kg) ^a^ | 30.4±5.3 | 29.5±5.5 | 28.1±5.0 | 27.8±5.1 | <0.001 |
| Minerals (kg) ^a^ | 2.9±0.5 | 2.7±0.5 | 2.6±0.4 | 2.6±0.5 | <0.001 |
| Cholesterol (mmol/L) ^a^ | 4.86±1.0 | 4.72±1.0 | 4.96±1.0 | 4.76±1.0 | <0.001 |
| HDL (mmol/L) | 1.34±0.4 | 1.29±0.4 | 1.38±0.4 | 1.25±0.3 | <0.001 |
| LDL (mmol/L) | 2.76±0.9 | 2.68±0.9 | 2.88±0.9 | 2.68±0.9 | <0.001 |
| Comorbidity (yes) | 540 (11.6%) | 80 (17.6%) | 97 (12.1%) | 18 (14.1%) | 0.002 |
| Coronary heart disease (yes) | 181 (3.9%) | 17 (3.7%) | 32 (4.0%) | 7 (5.5%) | 0.830 |
| Hypertension (yes) | 1153 (24.8%) | 128 (28.2%) | 211 (26.3%) | 35 (27.3%) | 0.340 |
| Diabetes mellitus (yes) | 352 (7.6%) | 50 (11.0%) | 43 (5.4%) | 9 (7.0%) | 0.004 |
| Stroke (yes) | 80 (1.7%) | 22 (4.8%) | 13 (1.6%) | 7 (5.5%) | <0.001 |
| Depression (yes) | 745 (16.0%) | 80 (17.6%) | 269 (33.7%) | 52 (40.6%) | <0.001 |
| Malnutrition risk (yes) | 815 (19.4%) | 84 (20.9%) | 394 (49.7%) | 70 (55.1%) | <0.001 |
| Fall during last one year (yes) | 398 (16.7%) | 63 (20.6%) | 96 (19.6%) | 25 (27.5%) | 0.016 |
| Hospitalization during last one year (yes) | 999 (21.5%) | 123 (27.1%) | 224 (28.0%) | 35 (27.3%) | <0.001 |

Notes: ^a^: continuous variables, shown as mean±SD; ^b^: categorical variables, shown as Number (%). Test: *Chi-Squared Test* and *ANOVA*; MCR: motoric cognitive risk syndrome; CI: cognitive impairment; SPPB: Short Physical Performance Battery, which is an assessment tool for evaluation of the low extremity functioning in older people. BMI: body mass index, calculated as weight in kilograms divided by height in meters squared. HDL: high-density lipoprotein; LDL: high-density lipoprotein; COPD: chronic obstructive pulmonary disease.

# Table S2. Distributions of demographic and clinical characteristics of motoric cognitive risk syndrome and cognitive impairment status.(n=577)

| Characteristics | Neither  (N=410) | MCR-only  (N=82) | CI-only  (N=66) | MCR-CI  (N=19) | *p* value |
| --- | --- | --- | --- | --- | --- |
| Age ^a^ | 67.39±9.22 | 69.04±6.5 | 73.47±8.81 | 74.16±9.22 | <0.001 |
| Female ^b^ | 265 (64.6%) | 48 (58.5%) | 51 (77.3%) | 10 (52.6%) | 0.067 |
| Marital status ^b^ |  |  |  |  | 0.253 |
| Married | 316 (77.1%) | 63 (76.8%) | 48 (72.7%) | 11 (57.9%) |  |
| Unmarried/Widowed/Divorced | 94 (22.9%) | 19 (23.2%) | 18 (27.3%) | 8 (42.1%) |  |
| Ethnicity ^b^ |  |  |  |  |  |
| Han | 410 (100%) | 82 (100%) | 66 (100%) | 19 (100%) |  |
| Education ^b^ |  |  |  |  | <0.001 |
| Illiterate | 113 (27.6%) | 24 (29.3%) | 41 (62.1%) | 10 (52.6%) |  |
| Primary school | 160 (39.0%) | 39 (47.6%) | 18 (27.3%) | 5 (26.3%) |  |
| Middle school | 93 (22.7%) | 13 (15.9%) | 4 (6.1%) | 2 (10.5%) |  |
| Secondary school and above  above | 44 (10.7%) | 6 (7.3%) | 3 (4.5%) | 2 (10.5%) |  |
| BMI ^a^ | 23.02±3.42 | 23.07±3.96 | 23.72±6.8 | 23.08±3.51 | 0.625 |
| Obese (yes)^b^ | 34 (8.3%) | 4 (4.9%) | 5 (7.6%) | 2 (10.5%) | 0.726 |
| Low handgrip (yes) ^b^ | 306 (74.6%) | 63 (76.8%) | 57 (86.4%) | 17 (89.5%) | 0.076 |
| 4-Meter gait speed (m/s) ^a^ | 0.82±0.27 | 0.49±0.1 | 0.7±0.21 | 0.44±0.12 | <0.001 |
| Skeletal Muscle Mass (kg) ^a^ | 19.28±3.53 | 19.57±4.26 | 18.35±3.96 | 18.84±3.93 | 0.201 |
| Body Fat Mass (kg) ^a^ | 17.72±6.04 | 18.07±5.9 | 16.47±5.23 | 15.58±5.97 | 0.155 |
| Total Body Water (kg) ^a^ | 26.76±4.38 | 27.1±5.28 | 25.69±4.91 | 26.51±4.9 | 0.272 |
| Minerals (kg) ^a^ | 2.53±0.36 | 2.56±0.45 | 2.46±0.39 | 2.55±0.43 | 0.517 |
| Cholesterol (mmol/L) ^a^ | 4.67±0.98 | 4.72±0.89 | 4.61±0.93 | 4.54±1.35 | 0.867 |
| HDL (mmol/L) | 1.31±0.31 | 1.31±0.34 | 1.32±0.31 | 1.21±0.2 | 0.568 |
| LDL (mmol/L) | 2.56±0.83 | 2.55±0.73 | 2.48±0.76 | 2.56±1.13 | 0.917 |
| Comorbidity (yes) | 59 (14.4%) | 17 (20.7%) | 8 (12.1%) | 4 (21.1%) | 0.371 |
| Coronary heart disease (yes) | 21 (5.1%) | 5 (6.1%) | 2 (3.0%) | 1 (5.3%) | 0.859 |
| Hypertension (yes) | 99 (24.1%) | 26 (31.7%) | 25 (37.9%) | 7 (36.8%) | 0.059 |
| Diabetes mellitus (yes) | 42 (10.2%) | 14 (17.1%) | 5 (7.6%) | 0 (0.0%) | 0.086 |
| Stroke (yes) | 10 (2.4%) | 2 (2.4%) | 1 (1.5%) | 2 (10.5%) | 0.167 |
| Depression (yes) | 54 (13.2%) | 12 (14.6%) | 39 (59.1%) | 6 (31.6%) | 0.003 |
| Malnutrition risk (yes) | 103 (25.1%) | 17 (20.7%) | 39 (59.1%) | 10 (52.6%) | <0.001 |
| Fall during last one year (yes) | 40 (9.8%) | 8 (9.8%) | 10 (15.2%) | 5 (26.3%) | 0.056 |
| Hospitalization during last one year (yes) | 103 (25.1%) | 24 (29.3%) | 18 (27.3%) | 4 (21.1%) | 0.823 |

Notes: ^a^: continuous variables, shown as mean±SD; ^b^: categorical variables, shown as Number (%). Test: *Chi-Squared Test* and *ANOVA*; MCR: motoric cognitive risk syndrome; CI: cognitive impairment; SPPB: Short Physical Performance Battery, which is an assessment tool for evaluation of the low extremity functioning in older people. BMI: body mass index, calculated as weight in kilograms divided by height in meters squared. HDL: high-density lipoprotein; LDL: high-density lipoprotein; COPD: chronic obstructive pulmonary disease.

# Table S3. The adjusted model for clinical characteristics associated with metabolic subtypes of motoric cognitive risk syndrome compared with the healthy.

| Characteristics  (OR, 95% CI) | MCR-I  (N=21) | MCR-II  (N=18) | MCR-III  (N=43) |
| --- | --- | --- | --- |
| BMI | 0.78 (0.68-0.89) ** | 0.86 (0.75-0.98) ** | 0.90 (0.83-0.98) ** |
| Grip strength value (kg) | 0.92 (0.86-0.98) ** | 0.94 (0.87-1.02) | 0.94 (0.90-0.99) ** |
| SPPB score | 0.56 (0.45-0.70) *** | 0.61 (0.49-0.76) *** | 0.64 (0.56-0.74) *** |
| Skeletal Muscle Mass (kg) | 0.73 (0.61-0.87) ** | 0.70 (0.5-0.86) ** | 0.82 (0.73-0.92) ** |
| Body Fat Mass (kg) | 0.89 (0.82-0.96) ** | 0.94 (0.87-1.02) | 0.98 (0.93-1.02) |
| Total Body Water (kg) | 0.78 (0.68-0.90) ** | 0.74 (0.63-0.88) ** | 0.85 (0.77-0.93) ** |
| Minerals (kg) | 0.24 (0.06-0.99) ** | 0.05 (0.01-0.27) ** | 0.21 (0.08-0.58) ** |
| Cholesterol (mmol/L) | 0.61 (0.37-1.02) | 0.91 (0.55-1.51) | 1.05 (0.78-1.43) |
| HDL (mmol/L) | 0.73 (0.21-2.62) | 0.26 (0.06-1.16) | 0.92 (0.40-2.13) |
| LDL (mmol/L) | 0.76 (0.48-1.21) | 0.66 (0.41-1.06) | 0.88 (0.63-1.25) |
| Comorbidity (yes) | 1.00 (0.29-3.47) | 0.66 (0.15-2.92) | 2.19 (1.10-4.38) ** |
| Hypertension (yes) | 1.18 (0.47-2.97) | 0.45 (0.13-1.56) | 1.42 (0.76-2.67) |
| Diabetes mellitus (yes) | 0.59 (0.08-4.45) | 3.18 (1.02-9.91) ** | 2.83 (1.33-6.04) ** |
| Malnutrition risk (yes) | 1.74 (0.63-4.80) | 0.83 (0.23-2.98) | 0.93 (0.42-2.05) |

Notes: Multiple logistic regression was applied compared with the healthy neither motoric cognitive risk syndrome nor cognitive impairment. The model had adjusted age and gender. *** p<0.001, ** p<0.05. P≤0.05 was considered significant. Abbreviations: BMI: body mass index; SPPB: Short Physical Performance Battery; HDL: high-density lipoprotein; LDL: low-density lipoprotein.

# Table S4. The 24 risk metabolites of MCR-only.

|  | m/z | OR (95% CI) | p value |
| --- | --- | --- | --- |
| Reduced NR | 257.11375 | 0.71 (0.55-0.90) | 0.0061 |
| 2,4-Dihydroxypteridine | 163.02563 | 0.78 (0.62-0.99) | 0.0383 |
| 3-Ketosphingosine | 298.27406 | 0.75 (0.58-0.97) | 0.0289 |
| 7-Methylguanine | 166.07234 | 0.77 (0.61-0.98) | 0.0368 |
| Asymmetric dimethylarginine | 203.15025 | 0.67 (0.51-0.88) | 0.0058 |
| Benzylamine | 108.08078 | 0.69 (0.53-0.89) | 0.0049 |
| D-Phenyl lactic acid | 167.07027 | 0.74 (0.58-0.94) | 0.0144 |
| Glu-Gly | 205.08245 | 0.75 (0.59-0.94) | 0.0141 |
| Glycochenodeoxycholic acid | 448.30685 | 1.29 (1.01-1.65) | 0.044 |
| L-Cystine | 241.03165 | 1.37 (1.06-1.79) | 0.0197 |
| L-Glutamic acid | 148.06096 | 1.30 (1.03-1.65) | 0.0297 |
| L-Proline | 116.07113 | 1.46 (1.15-1.85) | 0.0020 |
| N1-Acetylspermidine | 188.17574 | 0.69 (0.53-0.88) | 0.0041 |
| Vitamin B5 | 220.11795 | 0.76 (0.59-0.97) | 0.0331 |
| PC(30:3) | 700.49118 | 0.70 (0.53-0.91) | 0.0083 |
| PC(33:2) | 744.55378 | 0.77 (0.60-0.99) | 0.0458 |
| PC(37:4) | 796.58508 | 0.78 (0.61-0.98) | 0.0366 |
| PE(30:3) | 658.44423 | 0.74 (0.56-0.95) | 0.0213 |
| PE(38:3) | 770.56943 | 1.36 (1.05-1.78) | 0.0202 |
| SM(34:0) | 705.5905 | 0.76 (0.61-0.94) | 0.0115 |
| Cer(43:2) | 662.64457 | 0.75 (0.58-0.95) | 0.0213 |
| ChE(18:1) | 668.63401 | 0.78 (0.63-0.97) | 0.0240 |
| ChE(18:3) | 664.60271 | 0.77 (0.62-0.97) | 0.0213 |
| ChE(20:5) | 688.60271 | 0.77 (0.61-0.97) | 0.0243 |

Notes: The risk metabolites were screened by logistic correlation regression analysis. P-value represents the difference between MCR-only and Neither. Odds ratio (OR) and 95% confidence interval (95% CI) were displayed.

# Table S5. The 72 risk metabolites of CI-only.

|  | m/z | OR (95% CI) | p value |
| --- | --- | --- | --- |
| 12(S)PETE | 335.22278 | 0.58 (0.41-0.80) | 0.0012 |
| 13(S)POT | 309.20713 | 0.67 (0.49-0.89) | 0.0086 |
| 3a,7a,12a-Trihydroxy-5b-Cholestanoic acid | 451.3418 | 1.31 (1.02-1.70) | 0.0360 |
| Dihydroxycoprostane | 405.37271 | 0.73 (0.56-0.96) | 0.0219 |
| 3-Methyglutaric acid | 147.06571 | 0.71 (0.55-0.92) | 0.0086 |
| 3-Ydroxybenzoic acid | 137.0239 | 1.29 (1.01-1.65) | 0.0388 |
| Adenyl succinic acid | 462.06677 | 0.72 (0.53-0.96) | 0.0277 |
| Androstenedione | 287.20056 | 0.65 (0.49-0.85) | 0.0018 |
| Biliverdin | 583.25511 | 1.40 (1.06-1.85) | 0.0171 |
| Butyryl carnitine | 232.15433 | 1.36 (1.04-1.78) | 0.0251 |
| D-Glutamyl glycine | 203.06735 | 0.67 (0.50-0.89) | 0.0059 |
| Uric acid | 167.02054 | 0.74 (0.55-0.99) | 0.0435 |
| Uridine | 243.06174 | 0.72 (0.53-0.96) | 0.0262 |
| γ-Linolenyl carnitine | 422.32703 | 0.67 (0.51-0.88) | 0.0041 |
| Glu-Leu | 261.14505 | 0.67 (0.49-0.9) | 0.0091 |
| Glycine | 76.03983 | 0.76 (0.57-1.00) | 0.0497 |
| Hexylresorcinol | 193.1234 | 0.55 (0.40-0.73) | 0.0001 |
| L-Acetyl carnitine | 204.12303 | 1.40 (1.03-1.97) | 0.0426 |
| L-Aspartate | 132.02971 | 0.63 (0.46-0.86) | 0.0037 |
| Glu -Val | 247.1294 | 0.66 (0.49-0.86) | 0.0032 |
| Linoleic acid(18:2) | 279.23295 | 0.62 (0.45-0.83) | 0.0018 |
| Linoleyl carnitine | 424.34216 | 0.66 (0.50-0.88) | 0.0039 |
| L-Isoleucine | 132.10243 | 0.65 (0.49-0.86) | 0.0032 |
| L-Leucine | 132.10243 | 0.67 (0.51-0.88) | 0.0037 |
| L-Methionine | 150.05885 | 0.71 (0.54-0.92) | 0.0117 |
| LPE(16:1) | 450.26262 | 1.38 (1.04-1.82) | 0.0239 |
| L-Threonine | 120.06604 | 0.70 (0.53-0.91) | 0.0082 |
| L-Valine | 118.08678 | 0.73 (0.55-0.96) | 0.0252 |
| METHACHOLINE-like | 160.13321 | 0.76 (0.58-1.00) | 0.0500 |
| Methyl-L-histidine | 170.09293 | 0.67 (0.49-0.91) | 0.0122 |
| Mevalolactone | 131.07082 | 1.66 (1.18-2.36) | 0.0040 |
| Myristoleic acid(14:1) | 225.18546 | 1.36 (1.03-1.81) | 0.0301 |
| Ornithine | 133.09767 | 0.69 (0.51-0.91) | 0.0100 |
| Palmitoleic acid | 253.2173 | 1.55 (1.16-2.09) | 0.0033 |
| Vitamin B5 | 218.1034 | 0.68 (0.51-0.90) | 0.0086 |
| Pentadecanoic acid(15:0) | 241.2173 | 0.71 (0.51-0.95) | 0.0009 |
| PC(33:2) | 744.55378 | 0.60 (0.44-0.80) | 0.0280 |
| PC(34:2) | 744.59017 | 0.75 (0.57-0.97) | 0.0350 |
| PC(35:2) | 772.58508 | 0.75 (0.57-0.98) | 0.0066 |
| PC(37:2) | 800.61638 | 0.68 (0.52-0.90) | 0.0441 |
| PE(38:5) | 750.54322 | 0.75 (0.57-0.99) | 0.0314 |
| PE(38:8) | 746.51192 | 0.74 (0.56-0.97) | 0.0295 |
| SM(39:1) | 773.6531 | 0.71 (0.54-0.93) | 0.0121 |
| SM(41:1) | 801.6844 | 0.75 (0.57-0.97) | 0.0280 |
| SM(41:2) | 799.66875 | 0.77 (0.60-1.00) | 0.0498 |
| SM(41:4) | 795.63745 | 0.73 (0.56-0.95) | 0.0203 |
| SM(43:4) | 823.66875 | 0.76 (0.59-0.99) | 0.0400 |
| TG(50:1) | 850.78582 | 1.42 (1.07-1.91) | 0.0156 |
| TG(50:2) | 848.77017 | 1.71 (1.27-2.32) | 0.0004 |
| TG(50:3) | 846.75452 | 1.74 (1.28-2.4) | 0.0005 |
| TG(52:2) | 876.80147 | 1.43 (1.08-1.91) | 0.0126 |
| TG(48:2) | 820.73887 | 1.42 (1.07-1.91) | 0.0157 |
| TG(50:0) | 852.80147 | 1.36 (1.03-1.81) | 0.0327 |
| TG(52:1) | 878.81712 | 1.49 (1.11-2.02) | 0.0086 |
| TG(50:6) | 823.68102 | 1.40 (1.05-1.87) | 0.0224 |
| TG(52:7) | 849.69667 | 1.59 (1.19-2.15) | 0.0020 |
| Cer(40:0) | 624.62892 | 0.68 (0.51-0.90) | 0.0089 |
| Cer(40:1) | 622.61327 | 0.72 (0.54-0.94) | 0.0186 |
| Cer(41:1) | 636.62892 | 0.67 (0.5-0.88) | 0.0054 |
| Cer(41:2) | 634.61327 | 0.69 (0.51-0.91) | 0.0110 |
| Cer(42:0) | 652.66022 | 0.75 (0.56-0.99) | 0.0445 |
| Cer(42:1) | 650.64457 | 0.75 (0.57-0.99) | 0.0468 |
| FA(17:1) | 267.2328 | 1.37 (1.04-1.81) | 0.0284 |
| FA(20:2) | 307.2643 | 1.49 (1.11-2.01) | 0.0079 |
| FA(22:3) | 333.2804 | 1.45 (1.12-1.88) | 0.0051 |
| LysoPC(16:1) | 494.32466 | 1.46 (1.11-1.94) | 0.0081 |
| LysoPE(16:0) | 454.29336 | 1.45 (1.10-1.92) | 0.0086 |
| LysoPE(18:1) | 480.30901 | 1.32 (1.01-1.74) | 0.0435 |
| LysoPE(20:1) | 508.34031 | 1.34 (1.02-1.77) | 0.0372 |
| LysoPE(20:3) | 504.30901 | 1.52 (1.15-2.02) | 0.0031 |
| LysoPE(20:4) | 502.29336 | 1.49 (1.13-1.99) | 0.0057 |
| LysoPE(22:6) | 526.29336 | 1.54 (1.16-2.05) | 0.0031 |

Notes: The risk metabolites were screened by logistic correlation regression analysis. P-value represents the difference between CI-only and Neither. Odds ratio (OR) and 95% confidence interval (95% CI) were displayed.

# Table S6. The 45 risk metabolites of MCR-CI.

|  | m/z | OR (95% CI) | p value |
| --- | --- | --- | --- |
| Dihydroxycoprostane | 405.37271 | 0.64 (0.41-1.00) | 0.0442 |
| 3-Methyglutaric acid | 147.06571 | 0.64 (0.42-1.00) | 0.0452 |
| 3-Ydroxybutyric acid | 103.03955 | 0.56 (0.31-0.98) | 0.0469 |
| 4-Ydroxybenzaldehyde | 121.02898 | 1.66 (1.02-2.71) | 0.0401 |
| Alpha-Linolenic acid | 279.23186 | 0.61 (0.39-0.96) | 0.0310 |
| Capric acid(10:0) | 171.1385 | 0.57 (0.32-0.97) | 0.0463 |
| Creatine | 132.07728 | 0.54 (0.32-0.9) | 0.0173 |
| Delta-Tocopherol | 403.35706 | 1.76 (1.02-2.95) | 0.0363 |
| DL-Stearoyl carnitine | 428.37346 | 1.83 (1.10-3.15) | 0.0234 |
| Hexylresorcinol | 193.1234 | 0.53 (0.31-0.87) | 0.0155 |
| Linoleic acid(18:2) | 279.23295 | 0.53 (0.32-0.89) | 0.0161 |
| L-Leucine | 132.10243 | 0.53 (0.33-0.85) | 0.0085 |
| L-Threonine | 120.06604 | 0.62 (0.38-1.00) | 0.0482 |
| Pregnenolone | 315.23295 | 0.6 (0.42-0.87) | 0.0044 |
| PC(32:2) | 730.53813 | 0.49 (0.30-0.78) | 0.0032 |
| PC(33:2) | 744.55378 | 0.47 (0.28-0.77) | 0.0035 |
| PC(34:2) | 744.59017 | 0.57 (0.38-0.87) | 0.0087 |
| PC(35:2) | 772.58508 | 0.61 (0.37-0.96) | 0.0355 |
| PC(36:2) | 772.62147 | 0.57 (0.38-0.86) | 0.0068 |
| PC(36:3) | 770.60582 | 0.63 (0.40-0.98) | 0.0401 |
| PC(37:2) | 800.61638 | 0.53 (0.34-0.82) | 0.0040 |
| PC(37:5) | 794.56943 | 0.60 (0.38-0.93) | 0.0242 |
| PC(38:7) | 804.55378 | 0.60 (0.41-0.89) | 0.0082 |
| PE(36:4) | 724.52757 | 0.49 (0.28-0.81) | 0.0075 |
| PE(38:5) | 750.54322 | 0.48 (0.28-0.80) | 0.0056 |
| PE(38:6) | 750.54322 | 0.50 (0.30-0.81) | 0.0066 |
| PE(38:8) | 746.51192 | 0.46 (0.27-0.74) | 0.0021 |
| PE(40:6) | 776.55887 | 0.56 (0.34-0.89) | 0.0168 |
| PE(40:7) | 774.54322 | 0.57 (0.35-0.92) | 0.0223 |
| PE(42:10) | 798.54322 | 0.52 (0.32-0.84) | 0.0076 |
| SM(34:0) | 705.5905 | 0.64 (0.45-0.93) | 0.0108 |
| SM(39:1) | 773.6531 | 0.57 (0.36-0.88) | 0.0106 |
| SM(40:0) | 789.6844 | 0.66 (0.46-0.92) | 0.0170 |
| SM(40:1) | 787.66875 | 0.67 (0.45-1.01) | 0.0483 |
| SM(41:1) | 801.6844 | 0.59 (0.38-0.91) | 0.0175 |
| SM(41:2) | 799.66875 | 0.64 (0.42-0.97) | 0.0318 |
| SM(41:4) | 795.63745 | 0.59 (0.39-0.90) | 0.0128 |
| SM(43:4) | 823.66875 | 0.60 (0.39-0.91) | 0.0160 |
| SM(43:5) | 821.6531 | 0.66 (0.44-1.00) | 0.0454 |
| TG(50:2) | 848.77017 | 1.65 (1.01-2.76) | 0.0496 |
| TG(52:2) | 876.80147 | 1.96 (1.23-3.18) | 0.0053 |
| TG(52:3) | 874.78582 | 1.66 (1.03-2.75) | 0.0423 |
| Cer(40:1) | 622.61327 | 0.63 (0.39-0.99) | 0.0473 |
| Cer(41:1) | 636.62892 | 0.50 (0.29-0.82) | 0.0078 |
| Cer(41:2) | 634.61327 | 0.47 (0.27-0.80) | 0.0062 |

Notes: The risk metabolites were screened by logistic correlation regression analysis. P-value represents the difference between MCR-CI and Neither. Odds ratio (OR) and 95% confidence interval (95% CI) were displayed.

# Table S7. The 65 risk metabolites of MCR-I.

|  | m/z | OR (95% CI) | *p* value |
| --- | --- | --- | --- |
| 2,6-Dimethylheptanoyl carnitine | 302.23261 | 2.37 (1.20-5.54) | 0.0252 |
| 3-Ketosphingosine | 298.27406 | 0.32 (0.14-0.65) | 0.0035 |
| 4-Acetamidobutanoate | 146.08169 | 0.42 (0.17-0.89) | 0.0412 |
| 4-Pyrimidine methanamine | 110.07127 | 2.27 (1.21-4.74) | 0.0170 |
| Benzylamine | 108.08078 | 0.44 (0.18-0.89) | 0.0367 |
| Betaine | 118.08678 | 2.76 (1.34-6.62) | 0.0116 |
| Caproylcholine | 202.1807 | 2.27 (1.16-5.42) | 0.0316 |
| Hydrocinnamic acid | 149.06025 | 2.04 (1.08-4.34) | 0.0409 |
| L-Histidine | 156.07727 | 2.37 (1.25-5.01) | 0.0132 |
| Myristic acid(14:0) | 227.20111 | 0.45 (0.20-0.88) | 0.0296 |
| N-Acetylneuraminic acid | 310.11378 | 0.47 (0.22-0.90) | 0.0333 |
| Oleamide | 282.27914 | 0.41 (0.17-0.82) | 0.0227 |
| Ornithine | 133.09767 | 2.02 (1.08-4.08) | 0.0342 |
| Pentadecanoic acid(15:0) | 241.2173 | 0.40 (0.16-0.85) | 0.0293 |
| Cer(41:2) | 634.61327 | 0.49 (0.23-0.96) | 0.0480 |
| Cer(43:2) | 662.64457 | 0.44 (0.19-0.85) | 0.0246 |
| LysoPC(14:0) | 468.30901 | 0.42 (0.16-0.84) | 0.0385 |
| LysoPC(16:0) | 496.33994 | 0.36 (0.14-0.73) | 0.0136 |
| LysoPC(16:1) | 494.32466 | 0.51 (0.25-0.95) | 0.0448 |
| LysoPC(18:2) | 520.34031 | 0.51 (0.25-0.95) | 0.0438 |
| LysoPC(22:1) | 578.41856 | 2.02 (1.10-4.00) | 0.0306 |
| LysoPE(18:0) | 482.32466 | 0.41 (0.19-0.81) | 0.0167 |
| LysoPE(20:1) | 508.34031 | 0.34 (0.12-0.77) | 0.0304 |
| PC(30:0) | 706.53813 | 0.27 (0.10-0.58) | 0.0026 |
| PC(32:1) | 732.55378 | 0.39 (0.17-0.78) | 0.0136 |
| PC(32:2) | 730.53813 | 0.44 (0.20-0.86) | 0.0241 |
| PC(33:1) | 746.56943 | 0.43 (0.19-0.85) | 0.0237 |
| PC(34:4) | 754.53813 | 0.37 (0.16-0.75) | 0.0112 |
| PC(40:7) | 818.60582 | 2.25 (1.20-4.75) | 0.0194 |
| PC(42:8) | 858.60073 | 2.76 (1.35-6.76) | 0.0118 |
| TG(48:5) | 797.66537 | 0.29 (0.11-0.62) | 0.0048 |
| TG(48:4) | 816.70757 | 0.48 (0.23-0.92) | 0.0391 |
| TG(50:7) | 821.66537 | 0.49 (0.23-0.93) | 0.0421 |
| TG(47:1) | 808.73887 | 0.18 (0.05-0.46) | 0.0018 |
| TG(49:1) | 836.77017 | 0.25 (0.09-0.55) | 0.0021 |
| TG(49:2) | 834.75452 | 0.29 (0.11-0.62) | 0.0036 |
| TG(49:3) | 832.73887 | 0.39 (0.17-0.76) | 0.0118 |
| TG(44:2) | 764.67627 | 0.33 (0.13-0.70) | 0.0098 |
| TG(42:0) | 740.67627 | 0.09 (0.01-0.36) | 0.0059 |
| TG(44:0) | 768.70757 | 0.18 (0.05-0.47) | 0.0021 |
| TG(46:3) | 790.69192 | 0.39 (0.16-0.78) | 0.0169 |
| TG(46:0) | 796.73887 | 0.22 (0.08-0.51) | 0.0017 |
| TG(46:1) | 794.72322 | 0.20 (0.06-0.49) | 0.0025 |
| TG(48:1) | 822.75452 | 0.28 (0.11-0.59) | 0.0023 |
| TG(48:0) | 824.77017 | 0.24 (0.08-0.53) | 0.0020 |
| TG(50:1) | 850.78582 | 0.42 (0.20-0.81) | 0.0161 |
| TG(50:2) | 848.77017 | 0.49 (0.24-0.93) | 0.0377 |
| TG(51:1) | 864.80147 | 0.32 (0.13-0.64) | 0.0040 |
| TG(51:2) | 862.78582 | 0.43 (0.20-0.82) | 0.0168 |
| TG(46:2) | 792.70757 | 0.27 (0.09-0.62) | 0.0064 |
| TG(48:2) | 820.73887 | 0.32 (0.13-0.67) | 0.0064 |
| TG(48:3) | 818.72322 | 0.40 (0.17-0.80) | 0.0178 |
| TG(50:0) | 852.80147 | 0.34 (0.15-0.70) | 0.0066 |
| TG(52:1) | 878.81712 | 0.45 (0.20-0.87) | 0.0286 |
| TG(53:1) | 892.83277 | 0.42 (0.20-0.80) | 0.0134 |
| TG(42:1) | 738.66062 | 0.32 (0.12-0.69) | 0.0088 |
| TG(44:1) | 766.69192 | 0.22 (0.06-0.52) | 0.0036 |
| TG(54:3) | 902.81712 | 2.39 (1.20-5.49) | 0.0216 |
| TG(54:5) | 898.78582 | 2.14 (1.12-4.38) | 0.0261 |
| TG(56:5) | 926.81712 | 2.13 (1.07-4.64) | 0.0396 |
| TG(49:4) | 830.72322 | 0.49 (0.23-0.93) | 0.0396 |
| TG(58:6) | 952.83277 | 2.31 (1.11-5.56) | 0.0388 |
| TG(50:6) | 823.68102 | 0.41 (0.18-0.80) | 0.0165 |
| TG(56:4) | 928.83277 | 2.72 (1.29-6.85) | 0.0166 |
| TG(56:9) | 901.72797 | 2.26 (1.18-4.83) | 0.0208 |

Notes: The risk metabolites were screened by logistic correlation regression analysis. P-value represents the difference between MCR-I and None. Odds ratio (OR) and 95% confidence interval (95% CI) were displayed.

# Table S8. The 75 risk metabolites of MCR-II.

|  | m/z | OR (95% CI) | *p* value |
| --- | --- | --- | --- |
| 4-Hydroxyindole | 134.0601 | 0.42 (0.18-0.84) | 0.0263 |
| 4-Ydroxybenzenesulfonic acid | 172.9914 | 0.50 (0.24-0.95) | 0.0471 |
| 5,6-Indolequinone-2-carboxylic acid | 190.0146 | 0.42 (0.19-0.84) | 0.0211 |
| Acetyl-N-formyl-5-Methoxykynurenamine | 263.1037 | 0.12 (0.03-0.35) | 0.0007 |
| Adenylsuccinic acid | 462.0668 | 0.45 (0.18-0.93) | 0.0474 |
| D-Glutamyl glycine | 203.0674 | 0.50 (0.23-0.96) | 0.0488 |
| Guanidineacetic acid | 118.0616 | 0.36 (0.15-0.72) | 0.0084 |
| Heptanoic acid | 129.0916 | 0.40 (0.16-0.82) | 0.0222 |
| Hexylresorcinol | 193.1234 | 0.37 (0.16-0.73) | 0.0078 |
| Hippurate | 178.0504 | 0.48 (0.23-0.90) | 0.0304 |
| Hydrocinnamic acid | 149.0603 | 0.22 (0.07-0.53) | 0.0030 |
| Indole-5,6-quinone | 146.0248 | 0.42 (0.19-0.81) | 0.0159 |
| Indoxyl sulfate | 212.0018 | 0.36 (0.15-0.73) | 0.0089 |
| L-Cystathionine | 223.0752 | 0.35 (0.14-0.72) | 0.0099 |
| N1-Acetylspermidine | 188.1757 | 0.34 (0.14-0.70) | 0.0069 |
| p-Cresol sulfate | 187.0065 | 0.43 (0.19-0.85) | 0.0232 |
| Pentadecanoic acid(15:0) | 241.2173 | 0.36 (0.14-0.78) | 0.0188 |
| Sphinganine | 302.3054 | 0.39 (0.18-0.77) | 0.0102 |
| Taurocholic acid | 514.2844 | 2.08 (1.06-4.62) | 0.0479 |
| Cer(41:1) | 636.6289 | 0.43 (0.19-0.83) | 0.0192 |
| Cer(41:2) | 634.6133 | 0.40 (0.17-0.81) | 0.0185 |
| Cer(43:2) | 662.6446 | 0.36 (0.15-0.74) | 0.0127 |
| Cer(44:1) | 678.6759 | 0.50 (0.24-0.93) | 0.0364 |
| ChE(16:0) | 642.6184 | 0.49 (0.22-0.92) | 0.0492 |
| ChE(18:1) | 668.634 | 0.49 (0.25-0.90) | 0.0281 |
| ChE(18:2) | 666.6184 | 0.50 (0.25-0.92) | 0.0345 |
| ChE(18:3) | 664.6027 | 0.40 (0.18-0.77) | 0.0115 |
| ChE(20:3) | 692.634 | 0.42 (0.20-0.80) | 0.0143 |
| ChE(20:4) | 690.6184 | 0.33 (0.15-0.64) | 0.0025 |
| ChE(20:5) | 688.6027 | 0.36 (0.15-0.73) | 0.0085 |
| ChE(22:6) | 714.6184 | 0.43 (0.20-0.81) | 0.0141 |
| DG(36:3) | 636.5562 | 2.54 (1.26-5.81) | 0.0154 |
| DG(36:4) | 634.5405 | 2.24 (1.12-5.09) | 0.0338 |
| Hex1Cer(34:1) | 700.5722 | 0.32 (0.12-0.69) | 0.0088 |
| Hex1Cer(38:1) | 756.6348 | 0.45 (0.22-0.86) | 0.0219 |
| Hex1Cer(40:1) | 784.6661 | 0.36 (0.15-0.73) | 0.0104 |
| Hex1Cer(41:1) | 798.6818 | 0.15 (0.04-0.41) | 0.0018 |
| PC(33:1) | 746.5694 | 0.36 (0.15-0.72) | 0.0081 |
| PC(33:2) | 744.5538 | 0.25 (0.09-0.58) | 0.0035 |
| PC(34:2) | 744.5902 | 0.48 (0.24-0.91) | 0.0327 |
| PC(35:1) | 774.6007 | 0.16 (0.04-0.45) | 0.0028 |
| PC(35:2) | 772.5851 | 0.33 (0.12-0.71) | 0.0105 |
| PC(36:4) | 782.5694 | 0.31 (0.12-0.63) | 0.0038 |
| PC(36:5) | 780.5538 | 0.31 (0.13-0.67) | 0.0058 |
| PC(37:4) | 796.5851 | 0.15 (0.04-0.41) | 0.0019 |
| PC(37:5) | 794.5694 | 0.48 (0.22-0.91) | 0.0358 |
| PC(38:4) | 796.6215 | 0.41 (0.19-0.78) | 0.0128 |
| PC(38:7) | 804.5538 | 0.39 (0.17-0.77) | 0.0145 |
| PC(38:8) | 788.5589 | 0.52 (0.25-0.96) | 0.0473 |
| PC(40:7) | 818.6058 | 0.40 (0.17-0.76) | 0.0114 |
| PC(40:8) | 830.5694 | 0.47 (0.22-0.90) | 0.0362 |
| PC(44:6) | 876.6841 | 0.49 (0.24-0.93) | 0.0381 |
| PE(41:5) | 808.5851 | 0.42 (0.19-0.82) | 0.0186 |
| SiE(18:2) | 694.6497 | 0.43 (0.20-0.81) | 0.0173 |
| SM(41:1) | 801.6844 | 0.43 (0.18-0.87) | 0.0300 |
| SM(41:2) | 799.6688 | 0.42 (0.18-0.84) | 0.0239 |
| SM(41:3) | 797.6531 | 0.45 (0.20-0.89) | 0.0307 |
| SM(41:4) | 795.6375 | 0.44 (0.18-0.91) | 0.0472 |
| SM(43:2) | 827.7001 | 0.31 (0.10-0.75) | 0.0259 |
| SM(43:4) | 823.6688 | 0.42 (0.18-0.84) | 0.0251 |
| SM(43:5) | 821.6531 | 0.43 (0.19-0.84) | 0.0238 |
| TG(56:2) | 932.8641 | 2.90 (1.44-6.70) | 0.0056 |
| TG(58:1) | 962.911 | 2.36 (1.22-5.13) | 0.0170 |
| TG(58:2) | 960.8954 | 2.54 (1.29-5.65) | 0.0119 |
| TG(60:2) | 988.9267 | 2.14 (1.14-4.43) | 0.0246 |
| TG(56:1) | 934.8797 | 2.70 (1.36-6.12) | 0.0082 |
| TG(54:1) | 906.8484 | 2.21 (1.15-4.74) | 0.0255 |
| TG(54:2) | 904.8328 | 2.54 (1.26-6.05) | 0.0176 |
| TG(58:3) | 958.8797 | 2.44 (1.24-5.42) | 0.0157 |
| TG(58:4) | 956.8641 | 2.12 (1.12-4.50) | 0.0303 |
| TG(62:3) | 1014.942 | 1.92 (1.05-3.77) | 0.0419 |
| TG(58:6) | 952.8328 | 2.19 (1.13-5.09) | 0.0369 |
| TG(56:3) | 930.8484 | 2.51 (1.28-5.64) | 0.0130 |
| TG(60:3) | 986.911 | 2.23 (1.18-4.70) | 0.0203 |
| TG(62:4) | 1012.927 | 1.91 (1.05-3.86) | 0.0445 |

Notes: The risk metabolites were screened by logistic correlation regression analysis. P-value represents the difference between MCR-II and None. Odds ratio (OR) and 95% confidence interval (95% CI) were displayed.

# Table S9. The 74 risk metabolites of MCR-III.

|  | m/z | OR (95% CI) | *p* value |
| --- | --- | --- | --- |
| 2,5-Dihydroxybenzoic acid | 153.0193 | 0.40 (0.21-0.69) | 0.0021 |
| 4-Pyridoxic acid | 182.0454 | 0.59 (0.35-0.96) | 0.0396 |
| Arachidonic acid(20:4) | 303.233 | 0.56 (0.32-0.94) | 0.0365 |
| ChE(20:5) | 688.6027 | 0.53 (0.3-0.89) | 0.0230 |
| D-Glucuronolactone | 175.0243 | 2.10 (1.21-3.92) | 0.0129 |
| Eicosanoic acid(20:1) | 309.2794 | 0.55 (0.31-0.91) | 0.0252 |
| Glycine | 76.03983 | 0.57 (0.33-0.93) | 0.0317 |
| Hex1Cer(41:1) | 798.6818 | 1.69 (1.03-2.94) | 0.0461 |
| L-Glutamic acid | 148.061 | 1.87 (1.11-3.45) | 0.0280 |
| L-Proline | 116.0711 | 1.91 (1.11-3.66) | 0.0318 |
| L-Valine | 118.0868 | 1.75 (1.06-3.02) | 0.0329 |
| LysoPC(14:0) | 468.309 | 1.86 (1.09-3.42) | 0.0330 |
| LysoPC(18:2) | 520.3403 | 0.60 (0.35-0.98) | 0.0468 |
| LysoPE(20:3) | 504.309 | 1.84 (1.09-3.32) | 0.0296 |
| LysoPE(22:0) | 538.3873 | 0.55 (0.30-0.92) | 0.0303 |
| Methyl-D-galactoside | 193.0712 | 0.58 (0.33-0.96) | 0.0436 |
| Myristic acid(14:0) | 227.2011 | 1.91 (1.14-3.43) | 0.0200 |
| Myristoleic acid(14:1) | 225.1855 | 1.69 (1.03-2.96) | 0.0486 |
| N1-Acetylspermidine | 188.1757 | 0.50 (0.27-0.86) | 0.0183 |
| Oleamide | 282.2791 | 0.59 (0.35-0.97) | 0.0437 |
| Palmitic acid(16:0) | 255.233 | 1.83 (1.11-3.17) | 0.0218 |
| PC(32:1) | 732.5538 | 2.20 (1.26-4.15) | 0.0089 |
| PC(33:1) | 746.5694 | 1.72 (1.04-2.97) | 0.0416 |
| PC(34:4) | 754.5381 | 2.37 (1.36-4.45) | 0.0041 |
| PC(38:1) | 816.6477 | 0.56 (0.31-0.95) | 0.0395 |
| PC(38:7) | 804.5538 | 0.59 (0.34-0.97) | 0.0463 |
| PC(40:3) | 840.6477 | 0.46 (0.25-0.78) | 0.0069 |
| PC(40:7) | 818.6058 | 0.51 (0.28-0.87) | 0.0180 |
| PC(42:5) | 850.6684 | 0.48 (0.26-0.82) | 0.0117 |
| PC(44:5) | 878.6997 | 0.51 (0.28-0.88) | 0.0203 |
| PI(36:4) | 876.5597 | 1.78 (1.05-3.32) | 0.0482 |
| SM(32:1) | 675.5436 | 1.82 (1.08-3.34) | 0.0348 |
| SM(36:0) | 733.6218 | 2.17 (1.27-3.93) | 0.0068 |
| SM(38:3) | 755.6062 | 2.08 (1.20-3.87) | 0.0140 |
| SM(40:0) | 789.6844 | 1.99 (1.19-3.53) | 0.0121 |
| TG(46:5) | 797.6654 | 1.89 (1.15-3.32) | 0.0174 |
| TG(47:1) | 808.7389 | 1.79 (1.09-3.11) | 0.0282 |
| TG(49:1) | 836.7702 | 2.12 (1.26-3.86) | 0.0079 |
| TG(49:2) | 834.7545 | 2.26 (1.32-4.17) | 0.0050 |
| TG(49:3) | 832.7389 | 2.04 (1.21-3.66) | 0.0108 |
| TG(51:3) | 860.7702 | 1.79 (1.06-3.21) | 0.0361 |
| TG(44:2) | 764.6763 | 1.84 (1.12-3.18) | 0.0208 |
| TG(46:3) | 790.6919 | 1.79 (1.09-3.10) | 0.0271 |
| TG(46:1) | 794.7232 | 1.80 (1.1-3.12) | 0.0260 |
| TG(48:1) | 822.7545 | 1.83 (1.11-3.20) | 0.0229 |
| TG(48:0) | 824.7702 | 1.74 (1.07-2.96) | 0.0316 |
| TG(50:1) | 850.7858 | 1.96 (1.18-3.47) | 0.0135 |
| TG50:2) | 848.7702 | 2.68 (1.54-5.19) | 0.0013 |
| TG(50:3) | 846.7545 | 2.37 (1.44-4.33) | 0.0023 |
| TG(50:4) | 844.7389 | 1.75 (1.06-3.01) | 0.0324 |
| TG(51:1) | 864.8015 | 1.89 (1.14-3.32) | 0.0185 |
| TG(51:2) | 862.7858 | 2.26 (1.32-4.19) | 0.0052 |
| TG(56:2) | 932.8641 | 0.52 (0.29-0.87) | 0.0182 |
| TG(58:2) | 960.8954 | 0.56 (0.32-0.92) | 0.0272 |
| TG(60:2) | 988.9267 | 0.52 (0.29-0.87) | 0.0171 |
| TG(46:2) | 792.7076 | 1.97 (1.18-3.49) | 0.0129 |
| TG(48:2) | 820.7389 | 2.33 (1.37-4.34) | 0.0036 |
| TG(48:3) | 818.7232 | 1.96 (1.18-3.47) | 0.0135 |
| TG(50:0) | 852.8015 | 1.70 (1.04-2.89) | 0.0398 |
| TG(42:1) | 738.6606 | 1.71 (1.05-2.92) | 0.0377 |
| TG(44:1) | 766.6919 | 1.76 (1.07-3.03) | 0.0317 |
| TG(54:3) | 902.8171 | 0.51 (0.29-0.85) | 0.0135 |
| TG(54:5) | 898.7858 | 0.38 (0.20-0.67) | 0.0015 |
| TG(58:3) | 958.8797 | 0.43 (0.24-0.73) | 0.0034 |
| TG(58:4) | 956.8641 | 0.38 (0.20-0.67) | 0.0016 |
| TG(62:3) | 1014.942 | 0.47 (0.25-0.79) | 0.0084 |
| TG(54:7) | 894.7545 | 0.58 (0.33-0.97) | 0.0477 |
| TG(50:6) | 823.681 | 1.92 (1.16-3.36) | 0.0153 |
| TG(52:7) | 849.6967 | 1.71 (1.05-2.90) | 0.0357 |
| TG(56:3) | 930.8484 | 0.41 (0.21-0.71) | 0.0030 |
| TG(56:4) | 928.8328 | 0.53 (0.30-0.88) | 0.0207 |
| TG(60:3) | 986.911 | 0.48 (0.27-0.80) | 0.0079 |
| TG(56:9) | 901.728 | 0.44 (0.24-0.77) | 0.0068 |
| TG(62:4) | 1012.927 | 0.46 (0.23-0.79) | 0.0100 |

Notes: The risk metabolites were screened by logistic correlation regression analysis. P-value represents the difference between MCR-III and None. Odds ratio (OR) and 95% confidence interval (95% CI) were displayed.
