# Supplementary figures and images for: Plasma metabolomics and lipidomics signatures of motoric cognitive risk syndrome in community-dwelling older adults
Source: Front Aging Neurosci. 2022 Sep 7;14:977191. doi: 10.3389/fnagi.2022.977191 (PMC9490321; doi:10.3389/fnagi.2022.977191)

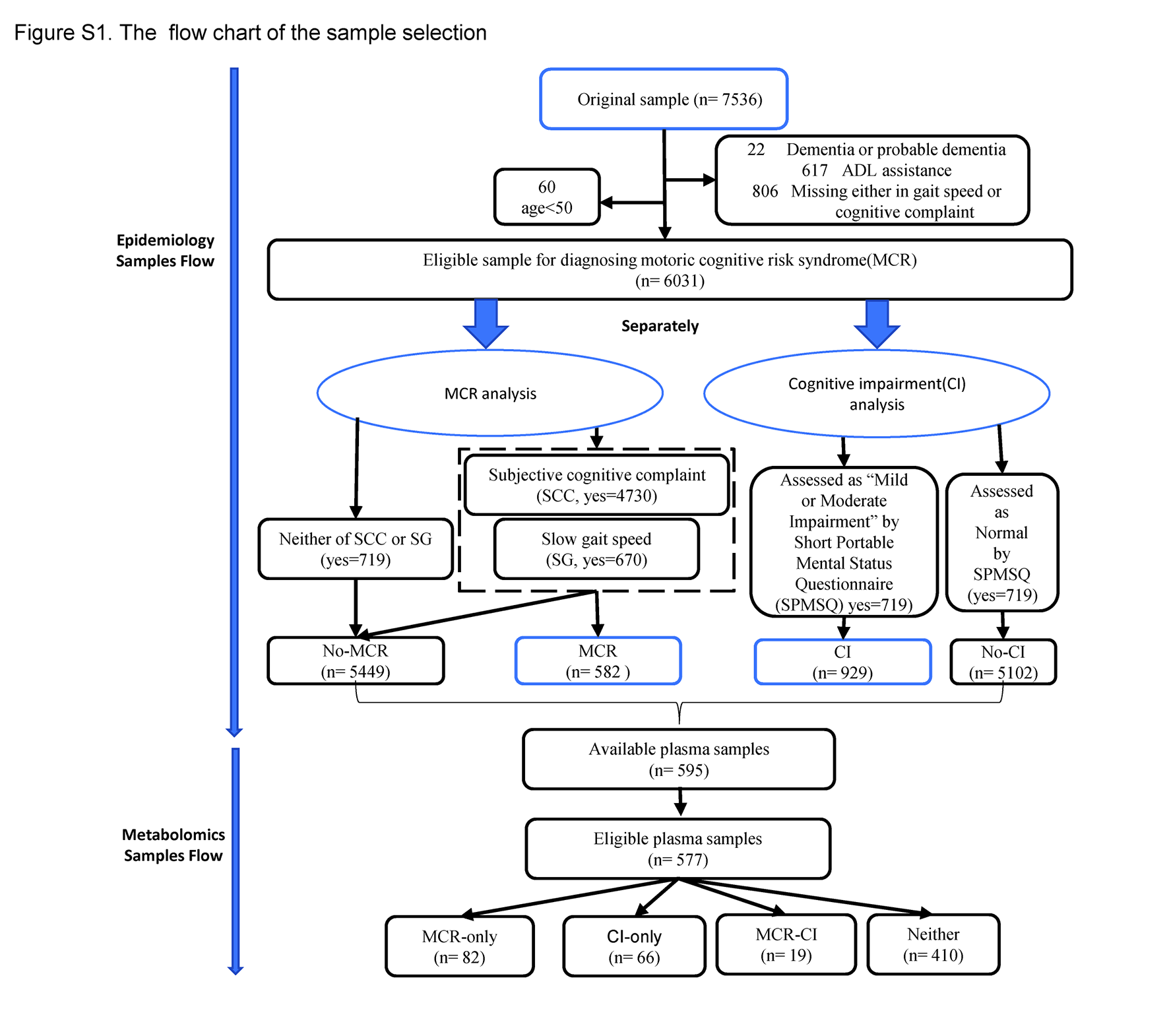

Supplement: Supplementary file 2 [file Image_1.TIF]

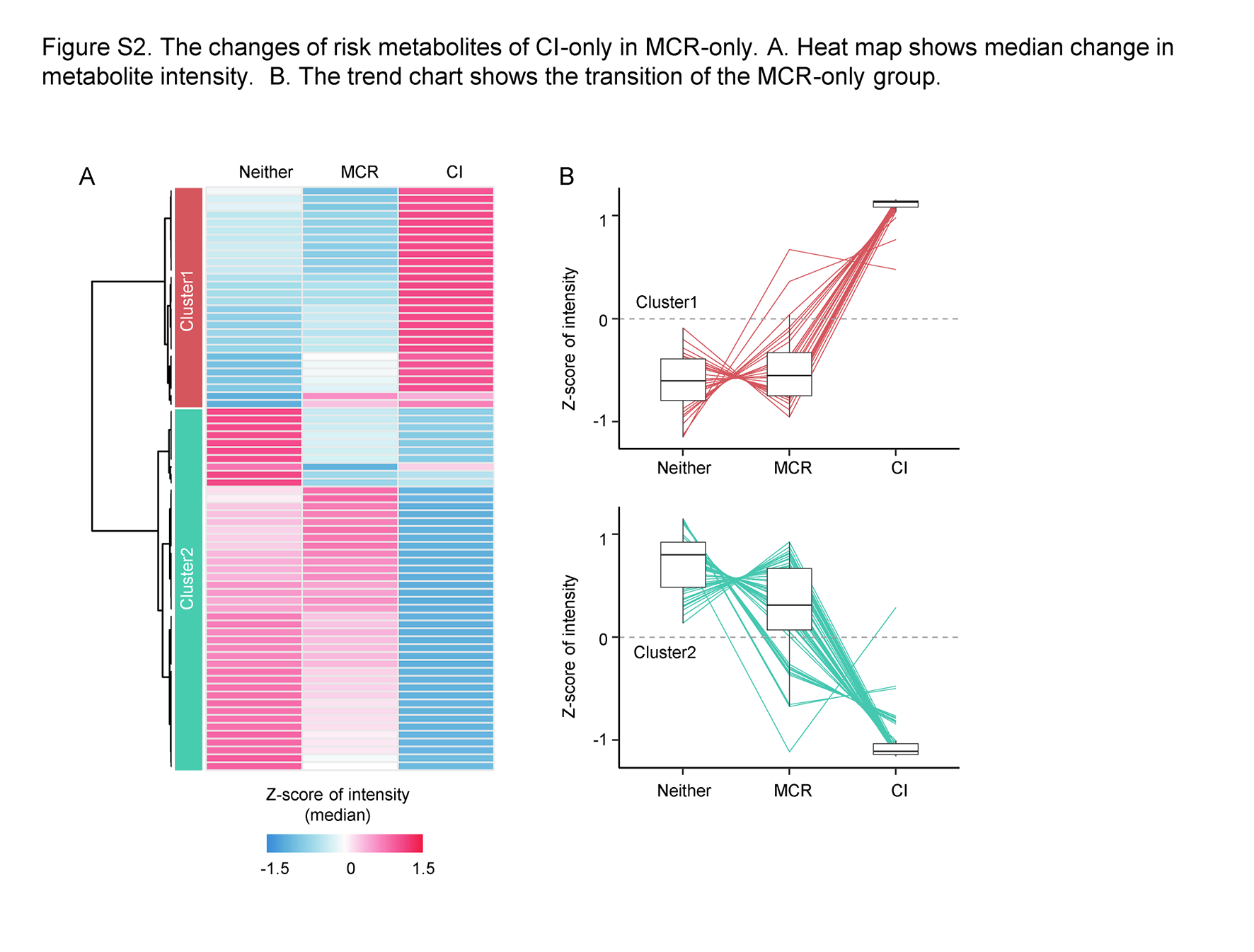

Supplement: Supplementary file 3 [file Image_2.TIF]
